# Tunable Electronic Properties of Graphene/g-AlN Heterostructure: The Effect of Vacancy and Strain Engineering

**DOI:** 10.3390/nano9121674

**Published:** 2019-11-23

**Authors:** Xuefei Liu, Zhaofu Zhang, Zijiang Luo, Bing Lv, Zhao Ding

**Affiliations:** 1 College of Big Data and Information Engineering, Guizhou University, Guiyang 550025, China; 201307129@gznu.edu.cn; 2 Semiconductor Power Device Reliability Engineering Center of Ministry of Education, Guiyang 550025, China; lah5200@sina.com; 3 Key Laboratory of Low Dimensional Condensed Matter Physics of Higher Educational Institution of Guizhou Province, School of Physics and Electronic Science, Guizhou Normal University, Guiyang 550025, China; binglv2816@sina.com; 4 Department of Engineering, University of Cambridge, Cambridge CB2 1PZ, UK; 5 College of Information, Guizhou Finance and Economics University, Guiyang 550025, China

**Keywords:** graphene/g-AlN heterostructure, Schottky barrier height, interface vacancy, biaxial strain, first-principles calculation

## Abstract

The structural and electronic properties of graphene/graphene-like Aluminum Nitrides monolayer (Gr/g-AlN) heterojunction with and without vacancies are systematically investigated by first-principles calculation. The results prove that Gr/g-AlN with nitrogen-vacancy (Gr/g-AlN-V_N_) is energy favorable with the smallest sublayer distance and binding energy. Gr/g-AlN-V_N_ is nonmagnetic, like that in the pristine Gr/g-AlN structure, but it is different from the situation of g-AlN-V_N_, where a magnetic moment of 1 μ_B_ is observed. The metallic graphene acts as an electron acceptor in the Gr/g-AlN-V_N_ and donor in Gr/g-AlN and Gr/g-AlN-V_Al_ contacts. Schottky barrier height $\Phi_{B,n}$ by traditional (hybrid) functional of Gr/g-AlN, Gr/g-AlN-V_Al_, and Gr/g-AlN-V_N_ are calculated as 2.35 (3.69), 2.77 (3.23), and 1.10 (0.98) eV, respectively, showing that vacancies can effectively modulate the Schottky barrier height. Additionally, the biaxial strain engineering is conducted to modulate the heterojunction contact properties. The pristine Gr/g-AlN, which is a p-type Schottky contact under strain-free condition, would transform to an n-type contact when 10% compressive strain is applied. Ohmic contact is formed under a larger tensile strain. Furthermore, 7.5% tensile strain would tune the Gr/g-AlN-V_N_ from n-type to p-type contact. These plentiful tunable natures would provide valuable guidance in fabricating nanoelectronics devices based on Gr/g-AlN heterojunctions.

## 1. Introduction

Benefiting from the superior electrical, optoelectronic, thermal, and mechanical properties, two-dimensional (2D) materials, such as graphene [1,2], transition metal dichalcogenides [3,4,5,6], phosphorene [7,8], carbon nitride [9,10], and III-Nitrides (III-N) [11,12,13,14,15], have been extensively theoretically or experimentally investigated during the past decades. Graphene as a 2D sp^2^-hybridized monolayer carbon structure, was successfully prepared in 2004 [1,2]. It exhibits a strong ambipolar electric field effect, such that electron and hole concentrations reach up to 10^13^/cm^2^ with room-temperature mobilities of ~10,000 cm^2^/V·s [1]. Additionally, graphene is well known for its other fascinating electronic and quantum transport properties, such as massless Dirac fermions, high carrier mobility, and an intriguing quantum Hall effect, which make it promising for nanoelectronics and devices [2].

However, the gapless nature of graphene restricts its applications in electronics and optoelectronics. Heterostructure, by constructing graphene with other 2D materials, is an effective method to broaden the application of graphene. Recently, many works have been reported, such as electrostatically created bipolar graphene heterojunction [16], graphene/MoS_2_ [17], as well as graphene/g-GaN [18,19]. Besides, Ahmad et al. used a modified Hummer’s method to obtain a photoconducting material based on the boron nitride-graphene oxide composite layer. They found that the confine element composition of boron, nitrogen, carbon, and oxygen showed excellent photoconduction [20]. These graphene-based van der Waals (vdW) heterostructures not only exhibit novel optoelectronic properties far beyond their individual components, but also preserve their intrinsic electronic properties due to the lack of dangling bonds and the weak electron coupling between sublayers [19]. Experimentally, ultrathin Aluminum Nitrides (AlN) nanosheet with a larger lattice constant as compared to its bulk-like wurtzite phase was successfully epitaxially grown [21]. 2D AlN few-layer sandwiched between the graphene and Si substrates was also confirmed this year [22]. These experimental results not only prove that 2D AlN has a promising application in optoelectronic field, but also indicate that it is of practical significance in the theoretical calculation of heterojunction based on 2D AlN. It is well known that the heterostructure properties can be tuned by defects, such as vacancies, which is inevitably introduced during the fabrication of materials. However, it is difficult to intentionally introduce accurate quantity of vacancies into 2D materials in experiments. Thus, the theoretical calculation stands out, which is important for accurately capturing the impact of defects on the material properties and, in turn, contributes to explaining the phenomena experimentally observed. Additionally, strain engineering is another significant method for tuning the heterostructure electronic properties, as reported recently [23,24,25]. In recent years, van der Waals heterojunctions have been extensively reported, both experimentally and theoretically, and proved to be a broad application prospect [26,27]. Very recently, Sciuto et al. have investigated the Fermi-level engineering for graphene by contacting it with bulk AlN rather than 2D AlN, and found that the Fermi-level can be tuned through the polarity and surface reconstruction of nitride [28]. However, to our best knowledge, neither the graphene/g-AlN heterojunction itself nor the modulation of defect or strain on its physical properties have been theoretically reported. Thus, a systematic investigation on the graphene/g-AlN van de walls heterostructure is desirable.

In this work, we systematically studied the graphene/AlN heterojunction properties by the first-principles calculation. Vacancies in AlN are considered to obtain a comprehensive understanding. It is found that the band structures of both graphene and g-AlN are preserved upon their contacts. All of the structures are thermodynamically stable with negative binding energy. The results show Gr/g-AlN, Gr/g-AlN-V_Al_, and Gr/g-AlN-V_N_ to be a p-type, p-type, and n-type Schottky contact, respectively. Furthermore, biaxial strain could effectively tune the contact type. The p-type contact of Gr/g-AlN would change into n-type under a negative biaxial strain and turn into ohmic under a positive biaxial strain. These important findings will provide valuable guidance for experimentalists to fabricate Gr/g-AlN-based devices.

## 2. Computational Details

The calculation was conducted based on the spin-polarized Kohn–Sham theory in the Perdew-Burke-Ernzerhof version of generalized gradient approximation (GGA-PBE) [29] with the projector augmented wave (PAW) potentials [30], as implemented in the VASP code [31,32]. The plane-wave cutoff was 450 eV and reciprocal space was sampled with a 5 × 5 × 1 Monkhorst-Pack k-point mesh. All of the atoms were relaxed until the Hellman–Feynman force on individual atoms less than 0.01 eV/Å and the total energy difference between two successive steps was lower than 10^−6^ eV. vdW correction and dipole correction were both considered in all calculations. A 20 Å vacuum slab was added to avoid interaction between adjacent images. The band structure analysis was conducted while using VASPKIT, a pre- and post-processing program for the VASP code [33]. The heterojunction binding energy is used to describe the relative stability of the heterostructure, as defined by Equation (1):

(1) $$E_{b}=E_{\mathrm{Gr}/\mathrm{AlN}}-\left(E_{\mathrm{Gr}}+E_{\mathrm{AlN}}\right)$$

where $E_{b}$ is the heterojunction binding energy; $E_{\mathrm{Gr}/\mathrm{AlN}}$ is the total energy of the heterostructure; and,$\;E_{\mathrm{Gr}}$ and $E_{\mathrm{AlN}}$ are the total energy of AlN and graphene monolayer, respectively. Negative $E_{b}$ means a stable heterostructure. The vacancy defect formation energy, which is important for predicting the concentration of certain defect, is defined as [34,35]:

(2) $$E_{\mathrm{vacancy}}^{f}\left[x\right]=E_{\mathrm{def}}\left[x\right]-E_{\mathrm{perfect}}-\sum n_{i}\mu_{i}$$

where $E_{\mathrm{def}}\left[x\right]$ is the total energy of a system containing a defect x; $E_{\mathrm{perfect}}$ represents the energy of a perfect supercell; $n_{i}$ is the number of atoms of atom x added (positive) or removed (negative) from the perfect system; and, $\mu_{i}$ is the atom’s chemical potential.

## 3. Results and Discussion

### 3.1. Sublayers and Heterostructures

**Figure 1:**
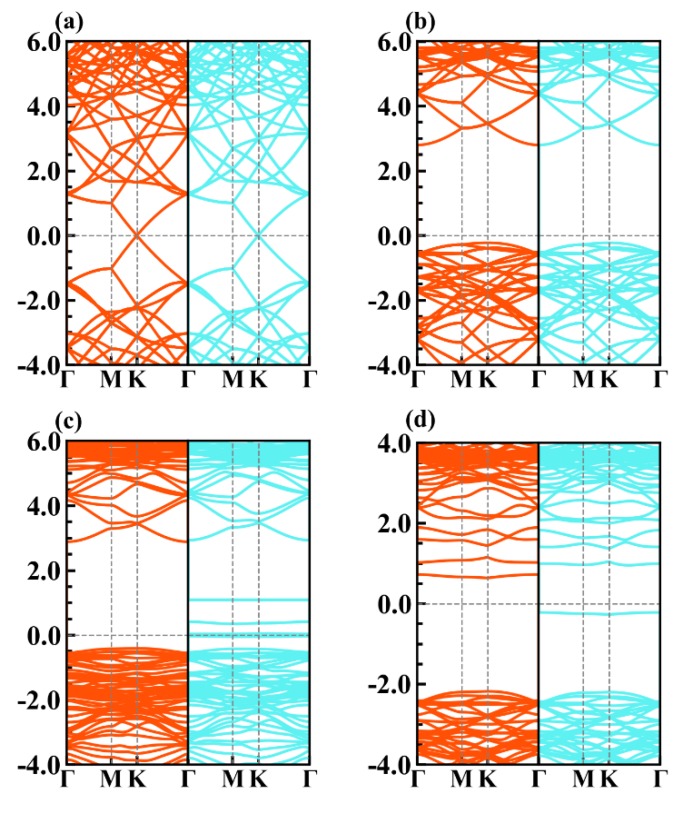
The band structures of (**a**) graphene, (**b**) g-AlN, (**c**) g-AlN-V_Al_, and (**d**) g-AlN-V_N_. The Fermi level is referred to zero energy. The spin-up and spin-down channels are marked with blue and red colors.

The relaxed lattice constants of graphene and AlN monolayers are 2.46 and 3.08 Å, respectively, as consistent with reports [36,37]. We first calculated the band structures of graphene, g-AlN, g-AlN monolayer with an aluminum vacancy (g-AlN-V_Al_) or a nitrogen vacancy (g-AlN-V_N_) to better compare the electronic differences of graphene and g-AlN monolayer before and after contacting, as shown in Figure 1. Graphene shows a zero-gap nature with an obvious Dirac cone (shown in Figure 1a). The g-AlN shows an indirect band gap of 3.07 eV while using the PBE functional (Figure 1b), close to that in our previous work [38]. The nonmagnetic nature of both graphene and g-AlN is found.

Several defect levels occur within the g-AlN-V_Al_ gap when an aluminum vacancy is introduced. Two of them are unoccupied and others are located just around the Fermi level (Figure 1c), indicating g-AlN-V_Al_ is a half-metal. The band gap of g-AlN-V_Al_ is slightly increased due to the nitrogen dangling bonds around the vacancy. In contrast, g-AlN-V_N_ is still a semiconductor, but with a magnetic moment of 1 μ_B_. The defect levels are found in both spin channels, only one of which is occupied in spin-up (right panel) channel locating ~0.25 eV lower than the Fermi level. Noting that the band gap of g-AlN-V_N_ is increased to ~3.4 eV due to the aluminum dangling bonds (Figure 1d). The electronic and magnetic properties of monolayer AlN with vacancy has been previously reported by us in detail [39].

**Figure 2:**
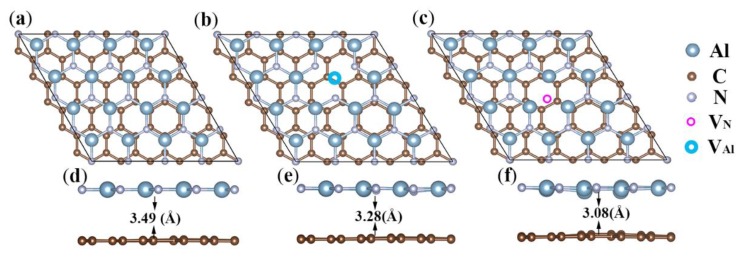
A schematic illustration of the Gr/g-AlN heterostructure with and without vacancy: (**a**) pristine Gr/g-AlN heterostructure, (**b**) Gr/g-AlN-V_Al_, and (**c**) Gr/g-AlN-V_N_. (**d**–**f**) are the corresponding side views with the layer distance labeled.

The Gr/g-AlN heterostructures are built while using the supercells with a lattice mismatch lower than 1%. The lattice constant of heterojunction is 12.33 Å, enlarged by four times and five times with respect to the primitive cell of g-AlN and graphene, respectively. Figure 2 shows the relaxed heterostructures.

The Gr/g-AlN structure without defects preserves a planar nature for both sublayers. In contrast, local distortion occurs when Al or N vacancy are introduced. The interlayer distance of pristine Gr/g-AlN is 3.49 Å and decreases to 3.28 (3.08) Å for Gr/g-AlN-V_Al_ (V_N_), all within the vdW gap range that was similar to previous reports [19,40]. A negative binding energy calculated by Equation (1) is found in all heterostructures, indicating that the Gr/g-AlN contacts are energetically stable. The heterostructure properties, including interlayer spacing (*d*), bond lengths of C-C ($L_{C-C}$), and Al-N ($L_{\mathrm{Al}-N})$ around vacancy, binding energy ($E_{b})\;$, gap, work function (WF), and Schottky barrier height (SBH) are listed in Table 1.

### 3.2. Electronic Properties

**Figure 3:**
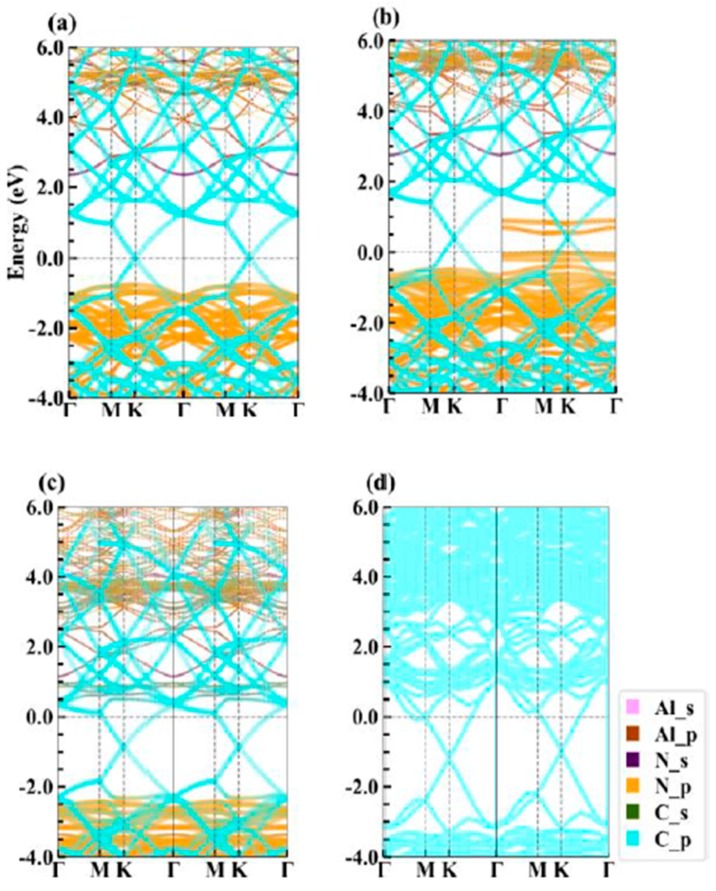
The band structures of (**a**) Gr/g-AlN, (**b**) Gr/g-AlN-V_Al_, and (**c**) Gr /g-AlN-V_N_. (**d**) is the HSE band structure of Gr/g-AlN-V_N_. Fermi level is set to zero energy. The spin-up and spin-down channels are plotted in the right and the left panels, respectively.

**Figure 4:**
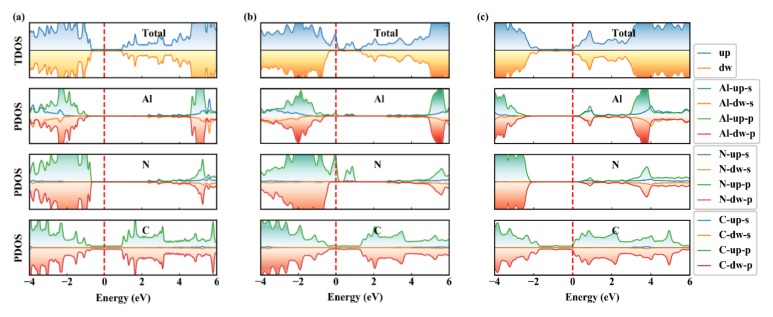
The projected density of states (PDOS) of (**a**) Gr/g-AlN, (**b**) Gr/g-AlN-V_Al_, and (**c**) Gr/g-AlN-V_N_ configurations, respectively. Fermi level is set to zero energy.

We calculate the projected band structures and projected density of states (PDOS) of Gr/g-AlN heterostructures to further investigate the electron properties, as plotted in Figure 3 and Figure 4, respectively. For comparison, the band structures of graphene, g-AlN, AlN with vacancies have already been shown in Figure 1.

The results in Figure 1a,b and Figure 3a prove little variation of the band structures of g-AlN and graphene before and after contacting, both with nonmagnetic nature. The results are similar to that of Gr/g-GaN [18,19], Gr/Sb [41], and Gr/MoSe_2_ heterojunctions [42]. The Fermi level exactly passes through the Dirac cone, which indicates that the charge transfer between graphene and g-AlN sublayers are negligible and barely affects the nature of graphene. Based on Equation (2), the formation energies of V_Al_ (under N-rich limitation) and V_N_ (under Al-rich limitation) are 8.14 and 3.16 eV, respectively. The positive formation energy means that it is hard to generate Al or N vacancy under the thermodynamic stability condition. However, Komsa et al. reported that vacancies can be produced by means of high-energy electron irradiation [43]. We expect that the same technology is applicable for artificially producing vacancies in the AlN monolayer. Only graphene could preserve the electronic properties when defects are introduced in g-AlN. Besides, the vacancy is inevitably induced in the high-temperature epitaxial growth chamber. The band gaps of the AlN sublayer in Gr/g-AlN, Gr/g-AlN-V_Al_, and Gr/g-AlN-V_N_ are calculated as 3.18, 3.13, and 3.14 eV, respectively. An increase of 0.11, 0.06, and 0.07 eV are obtained, respectively, when compared with the pristine g-AlN monolayer. Heyd-Scuseria-Ernzerhof (HSE) [44] functional was also used for comparison to have a more accurate gap value. The HSE band gaps of g-AlN sublayer in Gr/g-AlN, Gr/g-AlN-V_Al,_ and Gr/-g-AlN-V_N_ are 3.93, 3.98, and 4.11 eV, respectively. The HSE gap of freestanding g-AlN is 4.04 eV [38]. When vacancy defects are introduced, the band structures with defects are different from that of freestanding g-AlN and g-AlN in heterostructures, as shown in Figure 1c,d and Figure 3b,c.

The Gr/g-AlN-V_Al_ structure has a total magnetic moment of 3 μ_B_, as induced by symmetry-breaking in the vacant system, which agrees with our previous reported paper [39]. In Gr/g-AlN- $V_{\mathrm{Al}}\;$, two unoccupied defect levels in the spin-up channel are moved farther away from each other after contact with graphene. Some electrons are transferred to g-AlN from graphene, which leads the Dirac cone to shift above the Fermi level. Additionally, as seen in Figure 3b, the transferred electrons occupy the defect levels near the Fermi level, leading the states to become partially non-degenerated. However, the half-metal nature of g-AlN-V_Al_ is preserved, which is similar to the results of Gr/g-GaN in Ref. [19] and, therein, the results are also confirmed in Figure 4. The defect states located close to the valance band are mainly contributed by the N-p orbitals (Figure 4b), acting as an acceptor. The density of states (DOS) of Gr/g-AlN-V_Al_ near the Fermi level is very close to that of pristine Gr/g-AlN, where the PDOS of Al, N, and C hardly overlap with each other near the Fermi level. Thus, only weak interaction exists between the two sublayers. As a result, the interlayer distance (3.28 Å) is relatively larger than that of Gr/g-AlN-V_N_ (3.08 Å).

V_N_ can be more easily produced with a lower formation energy. Vacancies can induce gap states and magnetism, as shown in Figure 1b,d. By vertically contacting with graphene, the projected band structures of g-AlN sublayer in Gr/g-AlN-V_N_ (Figure 3c) are tuned back to resemble that in the freestanding g-AlN monolayer (Figure 1b). Thus, we expect that the growing g-AlN monolayer on graphene is beneficial in preserving its intrinsic nature, even N vacancy is unintentionally introduced. Additionally, the magnetic nature in freestanding g-AlN-V_N_ disappears after contacting graphene, which indicates that graphene could also tune the magnetism of g-AlN-V_N_, which shows potential application in electronic devices. The same phenomenon is also found by comparing the band structures of GaN-V_N_ in [45] and Gr/g-GaN-V_N_ in [18]. The disappearance of magnetism in Gr/g-AlN-V_N_ is because electrons occupied on the vacancy induced defect states would transfer to graphene, which leads the defect levels to shift up to conduction bands and then become unoccupied. As shown in Figure 3c, the Dirac cone of graphene decreases about 1eV and it is lower than the Fermi level, which confirms the electrons transfer from g-AlN to graphene. As a result, graphene acts as an acceptor in Gr/g-AlN-V_N_. The unoccupied states near the conduction band in Gr/g-AlN-V_N_ are mainly contributed by Al-s, Al-p, N-p, and C-p orbitals (Figure 4c). In addition, the PDOS of Al, N, and C atoms near the Fermi level have a similar shape, which results in strong orbital hybridization and interaction between the graphene and g-AlN sublayers. It agrees with the lowest interlayer distance (i.e., 3.08Å in Figure 2). 

The HSE band structure of Gr/g-AlN-V_N_ is shown in Figure 3d to further verify the results based on PBE functional, which is basically in accordance with that by PBE functional (Figure 3c), signifying that our other PBE band structures are also reliable. The reasons why we only use HSE functional to calculate Gr/g-AlN-V_N_ band structures are: (1) the defect formation energy of V_N_ is apparently lower than that of V_Al_, so it is more meaningful to discuss this more realistic contact in detail; (2) the band structures of Gr/g-AlN-V_N_ obviously change when compared with Gr/g-AlN, so it is necessary to verify the reliability of PBE results by using the more accurate HSE functional; (3) the HSE band structure calculation for such large supercells costs too much, while the core purpose in this work is to study how the vacancies and strain engineering would tune SBH, rather than evaluating the accurate defect level position. The CBM and VBM are confirmed to be at the Γ and K position, as that obtained with PBE functional. Noting that the VBM eigenvalue difference between location Γ and K is about ~0.5 eV, which is in agreement with that in Ref [14]. While considering this correction, we can obtain the HSE gaps as well as SBHs of Gr/g-AlN and Gr/g-AlN-V_Al_ by doing HSE self-consistent calculation, in which the Γ but not K point is included. This method is significantly more time saving than band calculation, and Table 1 lists the corresponding HSE results.

**Figure 5:**
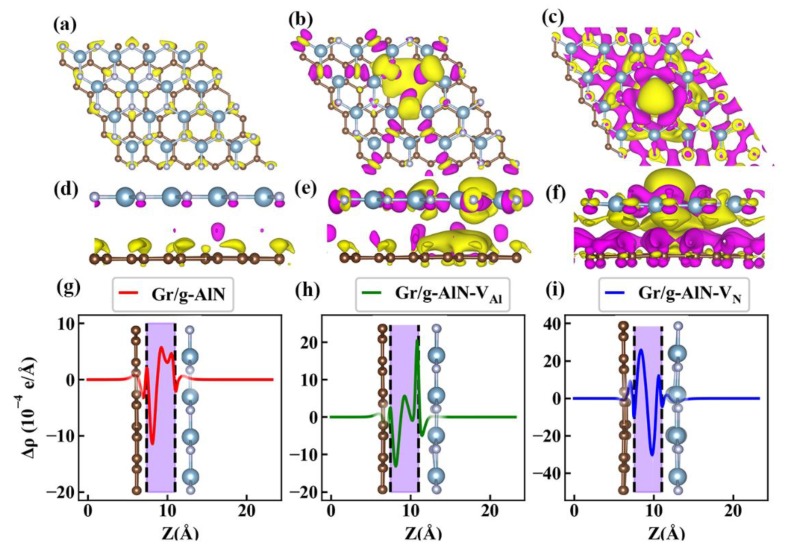
The iso-surface of differential charge density for (**a**) Gr/g-AlN, (**b**) Gr/g-AlN-V_Al_, and (**c**) Gr/g-AlN-V_N_, respectively. (**d**–**f**) are their corresponding side views. The purple and yellow color represent electron accumulation and depletion, respectively. The iso-surface is set to be $4\times 10^{-4}\;$e/Å^3^. Differential density Δρ along the *z*-direction for (**g**) Gr/g-AlN, (**h**) Gr/g-AlN-V_Al_, and (**i**) Gr/g-AlN-V_N._

The plane-averaged charge density difference (PCDD) between in Gr/g-AlN heterostructure can further describe the bonding nature and charge transfer, as defined by Equation (3):

(3) $$\Delta \rho =\rho_{\mathrm{Gr}/\mathrm{AlN}}-\rho_{\mathrm{AlN}}-\rho_{\mathrm{Gr}}$$

where $\rho_{\mathrm{Gr}/\mathrm{AlN}}$, $\rho_{\mathrm{AlN}}$, and $\rho_{\mathrm{Gr}}$ are the plane-averaged charge density of the Gr/g-AlN heterostructure, g-AlN monolayer and graphene monolayer, respectively. The PCDD clearly shows the interaction and electron transfer in heterostructures. It can be observed from Figure 5 that charge accumulation mainly occurs around the nitrogen atoms in the g-AlN region for Gr/g-AlN. In Gr/g-AlN-V_Al_, one can find that some electrons in graphene near aluminum vacancy would be depleted and then transform to g-AlN. While the graphene sublayer is found to act as an electron acceptor for Gr/g-AlN-V_N_.

Additionally, a better vision on the PCDD results (not shown here) shows that electrons around nitrogen are mainly contributed by p_z_-like orbitals in Gr/AlN and Gr/AlN-V_N_, while contributed by p_x(y)_-like orbitals in Gr/AlN-V_Al_, and the electrons that accumulated in graphene of Gr/AlN-V_N_ are found to be mainly occupying the p_z_-like orbitals. The results are consistent with their band structures in Figure 3 and are confirmed in Figure 5g,h.

**Figure 6:**
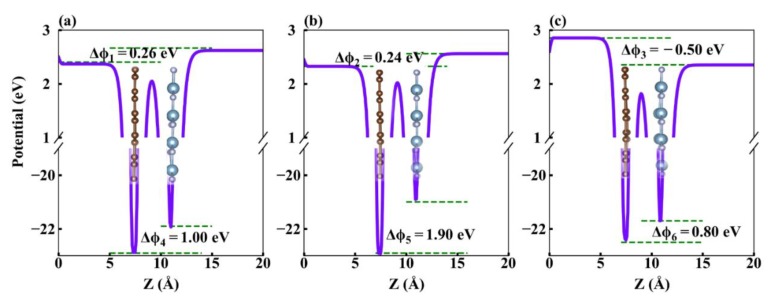
The planar averaged potential as a function of vacuum thickness in *z*-direction: (**a**) for pristine Gr/g-AlN, (**b**) for Gr/g-AlN-V_Al_, and (**c**) for Gr/g-AlN-V_N_. The graphene sublayer is located at the right side and g-AlN sublayer at left.

Figure 6 presents the planar averaged potential along the *z*-direction of Gr/g-AlN heterostructures. The energy difference in the vacuum regions for Gr/g-AlN and Gr/g-AlN-V_Al_ (V_N_) are 0.26 and 0.24 (−0.50 eV) (Δ$\Phi_{i}\;$, *i* = 1, 2, 3), indicating charge transfer and dipole formation at the interface. The potential differences in the sublayer regions are 1.00 and 1.90 (0.80) eV (shown in Figure 6 as Δ$\Phi_{i}\;$, *i* = 4, 5, 6), respectively. The results prove that V_Al_ would increase the potential difference, while $V_{N}$ would decrease it.

The work function WF is defined as: $\mathrm{WF}=E_{\mathrm{vac}}-E_{F}$, where $E_{\mathrm{vac}}$ and $E_{F}$ are the vacuum energy and Fermi energy, respectively. The work function for graphene, g-AlN, g-AlN-V_Al_, g-AlN-V_N_, Gr/g-AlN, Gr/g-AlN-V_Al_, and Gr/g-AlN-V_N_ under strain-free condition are 4.26, 5.10, 5.44, 3.29, 4.41, 4.80, and 3.27 eV, respectively, and the work function of graphene agrees with that in Ref. [18]. The differences in WF between graphene and g-AlN, g-AlN-V_Al_, and g-AlN-V_N_ are −0.84, −1.18, and 0.97 eV, respectively, once again proving that graphene would lose electrons (donor) in Gr/g-AlN and Gr/g-AlN-V_Al_, while obtaining electrons (acceptor) in Gr/g-AlN-V_N_.

### 3.3. Schottky Barrier Height

The Schottky barrier height, which is the energy difference between semiconductor band edges and metal Fermi level, is determined as in Equations (4) and (5):

(4) $$\Phi_{B,n}=\mathrm{CBM}-E_{F}$$

(5) $$\Phi_{B,p}=E_{F}-\mathrm{VBM}$$

where $\Phi_{B,n}$ is the n-type SBH, $\Phi_{B,p}$ is the p-type SBH, CBM is the conduction band minimum (CBM), VBM is the valence band maximum (VBM), and $E_{F}$ is the Fermi level, which is aligned to zero in this calculation. According to Figure 3a and Figure 4a, $\Phi_{B,n}$ and $\Phi_{B,p}$ of Gr/g-AlN are 2.35 and 0.83 eV, respectively. Accordingly, a p-type Schottky contact is formed at the Gr/g-AlN interface. Moreover, $\Phi_{B,n}$ and $\Phi_{B,p}$ are 2.77 eV and 0.36 eV for Gr/g-AlN-V_Al_, respectively, also a p-type contact. However, the SBHs are obviously changed by aluminum vacancy. In the case of Gr/g-AlN-V_N_, it is n-type contact, owing to a 1.10 eV $\Phi_{B,n}\;$. By band gap correcting with HSE functional, the $\Phi_{B,p}\;$($\Phi_{B,n})$ of Gr/g-AlN, Gr/g-AlN-V_Al_, and Gr/g-AlN-V_N_ are 0.24 eV (3.69 eV), 3.23 eV (0.75 eV), and 3.13 eV (0.98 eV), respectively. The HSE results are in compliance with the PBE counterparts, indicating our PBE-level calculations are qualitatively reliable. These results prove that vacancies can tune the SBH of Gr/g-AlN heterojunctions. Table 1 also summarizes the SBH data calculated with both PBE and HSE functionals.

### 3.4. Effects of Biaxial Strain on SBH

**Figure 7:**
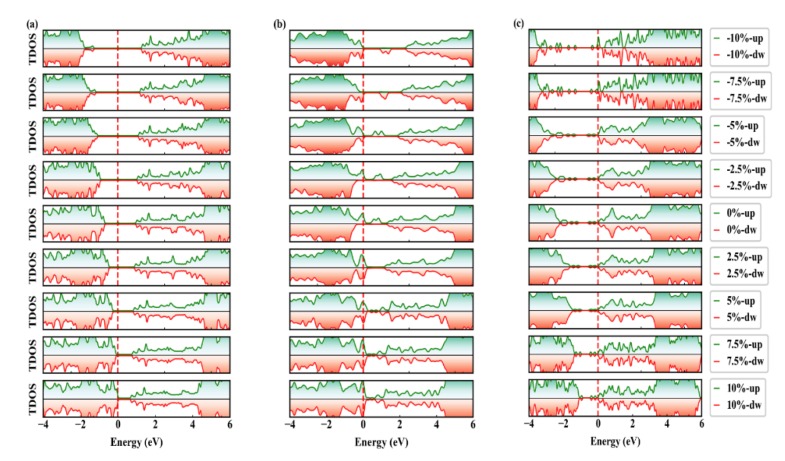
The PDOS for (**a**) Gr/g-AlN, (**b**) Gr/g-AlN-V_Al_, and (**c**) Gr/g-AlN-V_N_ under −10% to 10% biaxial strain. The green and red colors present the spin-up and spin-down channels, respectively. The Fermi level is set to zero energy.

**Figure 8:**
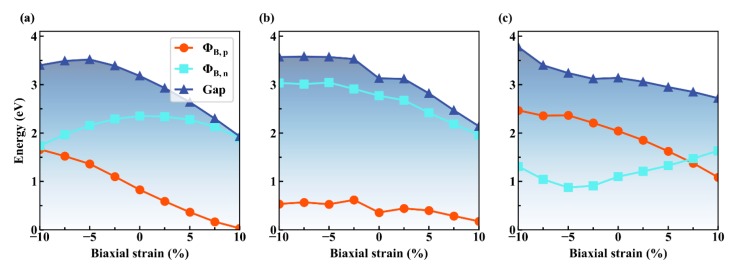
The evolution of the SBH as a function of the biaxial strain for (**a**) Gr/g-AlN, (**b**) Gr/g-AlN-V_Al_, and (**c**) Gr/g-AlN-V_N_. The red, cyan, and deep blue color represent the $\Phi_{B,n}\;$, $\Phi_{B,p}$, and the AlN gap value in heterostructures, respectively.

The Schottky barrier height can be effectively tuned by in-plane strain engineering [24], vertical strain engineering [46,47], and external electric field [48], etc. In this work, we focus on the impact of in-plane biaxial strain engineering on the SBHs of Gr/g-AlN heterostructures, which is theoretically preferable for flexible device applications. By applying biaxial (in both *x*- and *y*- directions) strain on the heterojunctions, the AlN band gap in Gr/g-AlN increases with the biaxial strain, changing from −10% to −5%, and it would decrease from −5% to 10%, reaching the maximum band gap with −5% strain. In contrast, the band gap of g-AlN in Gr/AlN-V_Al_ changes little when the compressive biaxial strain is larger than 2.5% and decreases within the range of −2.5% to 10%. For Gr/g-AlN-V_N_, the AlN band gap almost monotonously decreases within the whole strain range, as shown by the total DOS in Figure 7c and the triangle-line in Figure 8c.

These results are similar to the trend in MX_2_ monolayer [49], as well as that in Gr/g-GaN heterostructure [18] and Gr/MoSe_2_ heterostructure [42]. It is also observed in Figure 7 that the Fermi level shifts close to the VBM and away from the CBM for Gr/g-AlN and Gr/g-AlN-V_Al_, and an inverse phenomenon is found for Gr/g-AlN-V_N_.

Figure 8 summarizes the SBHs as a function of strain engineering. The strain is applied in the range from −10% to 10% of its fully relaxed lattice constant. When considering the computational expenses, all of the strained calculations are calculated with PBE functional, but these results would be qualitatively similar to that by HSE calculation based on the comparison under strain-free cases. Obviously, Gr/g-AlN shows a p-type contact in a large strain range.

With the increase of tensile strain, the $\Phi_{B,p}$ almost linearly decreases, owing to the combination of the decrease of g-AlN band gap, the nearly unchanged CBM, and the up-shifted VBM. While in the positive strain region, the Fermi level gradually moves close to and finally merges into the valance band, i.e., ohmic contact [6]. However, noting that such a large strain is not realistic in reliable device applications. The main purpose here is to grasp the variation trend and provide experimental guidance. Obviously, the $\Phi_{B,p}$ is always lower than $\Phi_{B,n}$ for Gr/g-AlN-V_Al_, even within a large strain range. Thus, it is the p-type Schottky contact with a stable SBH value of ~0.5 eV, despite the variations of strain.

These results mean that aluminum vacancy is beneficial for stabilizing the contact type in Gr/g-AlN, even if external stress is introduced. While for Gr/g-AlN-V_N_, it is n-type contact. By applying a 7.5% tensile biaxial strain, it transforms from the n-type back into p-type contact. The discussions on the strain engineering provide theoretical guidance on Gr/g-AlN based flexible device applications.

**Figure 9:**
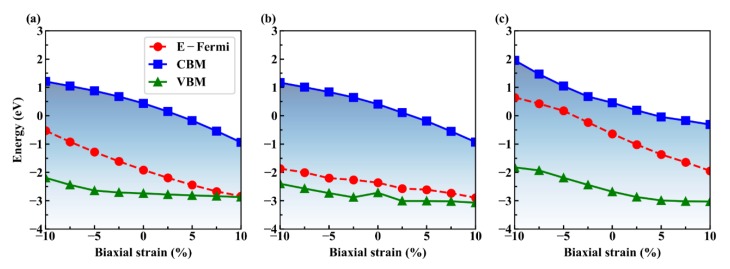
The conduction band minimum (CBM), valence band maximum (VBM) of AlN monolayer in Gr/g-AlN contact, and Fermi level as a function of applied strain for (**a**) Gr/g-AlN, (**b**) Gr/g-AlN-V_Al_, and (**c**) Gr/g-AlN-V_N_, respectively.

We plot the CBM, VBM, and the Fermi levels as a function of applied strain in Figure 9 in order to understand the variations of the SBH with strain in the Gr/g-AlN heterostructures. For Gr/g-AlN, the VBM is changed within the strain range from −10% to −5% and it nearly keeps constant after that, while the Fermi level and CBM decrease with a different speed in the corresponding strain range. This results in the $\Phi_{B,n}$ rising first and descending later, while the ${\;\Phi }_{B,p}$ monotonously decreases within the whole strain range in Figure 8a. For Gr/g-AlN-V_Al_, the VBM and Fermi level both change slightly with the change of strain, while the CBM decreases fast, which leads to the decrease of $\Phi_{B,n}$ in Figure 8b. In the case of Gr/g-AlN-V_N_, CBM, VBM, and Fermi level decrease at a different speed (Figure 9c), which leads to the Fermi level moving close to the CBM. As a result, contact-type transition occurs at a tensile strain of ~5% (Figure 8c).

## 4. Conclusions

In conclusion, we have systemically investigated the structural and electronic properties of Gr/g-AlN heterojunctions with and without vacancies by the first-principles methods. The Gr/g-AlN structure without defects preserves a planar nature for both sublayers. In contrast, local distortion occurs when Al or N vacancy is introduced. Gr/g-AlN-V_N_ is energy favorable, with the lowest binding energy of -2.90 eV and the smallest sublayer distance of 3.08 Å. Based on the results of the projected band structure and PDOS, we find the bandgap of g-AlN increasing slightly after contacting with graphene, and two unoccupied defect levels in the spin-up channel of Gr/g-AlN- $V_{\mathrm{Al}}$ are moved farther away from each other after contacting with graphene. Graphene is found to act as a weak electron donor in Gr/g-AlN and Gr/g-AlN-V_Al_, and acceptor in Gr/g-AlN-V_N_ heterostructure based on the charge transfer analysis. Besides, the magnetic nature in freestanding g-AlN-V_N_ disappears after contacting graphene, which indicates that graphene could tune the magnetism of g-AlN-V_N_. The results prove that the vacancy in g-AlN would strengthen the heterostructure interaction. Finally, the results show that Schottky barrier height can be effectively modulated by applying biaxial strain. Under the free-strain condition, Gr/g-AlN is found to be a p-type Schottky contact with a $\Phi_{B,p}$ of 0.83 eV and transform into an n-type contact by introducing a nitrogen vacancy. In contrast, aluminum vacancy would enhance the stability of the contact type of Gr/g-AlN under external strain. Our results can provide some trend-guidance for experimentalists, especially for those who want to modify the device characteristics by tuning the Schottky barrier. More specifically, our study is expected to promote the application of ultrathin Gr/g-AlN heterostructures that are based nanoelectronics devices with transparent and flexible nature, such as electric field effect transistor, tunneling transistor, Schottky devices, and so on [50,51].

## Figures and Tables

**Table 1:** Interlayer distance (*d*), bond length (*L*) around vacancy, binding energy ($E_{b}$), work function (WF), bandgap of AlN (or AlN in Gr/g-AlN), and Schottky barrier height (SBH). The Gap and SBH calculated by both Perdew-Burke-Ernzerhof (PBE) functional and Heyd-Scuseria-Ernzerhof (HSE) functional are shown for comparison.

Structures	*d* (Å)	$L_{C-C}\;(Å)$	$\;L_{Al-N}\;(Å)$	Gap (eV)		$\;E_{b}\;(\mathrm{eV})$	WF (eV)		SBH (eV)			
				PBE	HSE		PBE	HSE	$\Phi_{B,n}$		$\Phi_{B,p}$	
									PBE	HSE	PBE	HSE
Graphene	-	1.43	-	-	-	-	4.26	-	-	-	-	-
g-AlN	-	-	1.78	3.07	-	-	5.10	-	-	-	-	-
g-AlN-V_Al_	-	-	1.80	3.15	-	-	5.44	-	-	-	-	-
g-AlN-V_N_	-	-	1.81	3.40	-	-	3.29	-	-	-	-	-
Gr/g-AlN	3.49	1.42	1.78	3.18	3.93	−2.15	4.41	5.67	2.35	3.69	0.83	0.24
Gr/g-AlN-$V_{\mathrm{Al}}\;$	3.28	1.42	1.78	3.13	3.98	−2.89	4.80	5.12	2.77	3.23	0.36	0.75
Gr/g-AlN-$V_{N}\;$	3.08	1.43	1.78	3.14	4.11	−2.90	3.27	3.16	1.10	0.98	2.04	3.13
